# A systematic review of school meal nudge interventions to improve youth food behaviors

**DOI:** 10.1186/s12966-020-00983-y

**Published:** 2020-06-19

**Authors:** Jessica Jarick Metcalfe, Brenna Ellison, Nader Hamdi, Rachel Richardson, Melissa Pflugh Prescott

**Affiliations:** 1 Department of Food Science and Human Nutrition, University of Illinois at Urbana-Champaign, 905 South Goodwin Avenue, Urbana, IL 61801 USA; 2 Department of Agricultural and Consumer Economics, University of Illinois at Urbana-Champaign, 1301 West Gregory Drive, Urbana, IL 61801 USA

**Keywords:** Behavioral economics, School meals, Food waste, Nudge

## Abstract

**Background:**

School meal programs have a large reach and thus are ideal environments in which to implement interventions targeting improved youth eating behaviors and reduced food waste. This systematic review summarizes the evidence on the effectiveness of school meal nudge interventions on influencing children’s eating and waste behaviors.

**Methods:**

Inclusion criteria required studies have participants in primary or secondary school (grades K-12) with interventions that occurred during school lunch or breakfast in the cafeteria and included at least one of the following outcomes: selection, consumption, waste, or school meal participation. Analyses of intervention outcomes were restricted to studies of strong and moderate quality.

**Results:**

Twenty-nine studies were included in the quality assessment. Included interventions fell into three categories: 1) placement/convenience, 2) marketing/promotion, or 3) variety/portions. The 20 strong and moderate quality studies included in outcome analyses generally used strong data collection methods and study designs, but were limited by an overall lack of intervention fidelity checks. Multi-component interventions often did not use methods that allowed for separate analyses of outcomes for different intervention components.

**Conclusions:**

School meal nudge interventions were positively associated with food selection, and had an inconsistent relationship with food consumption. There were few studies evaluating the impact of nudge interventions on meal participation or food waste. The limited evidence available links nudges to improved meal participation, as well as undesirable increases in food waste. Future research in this area should use methods that incorporate implementation metrics, attend to systems factors, and allow the outcomes of individual intervention components to be isolated.

## Background

About 95% of U.S. children do not meet the federal dietary recommendations for vegetable intake [1, 2]. Concurrently, food waste is increasingly recognized as a major concern across the global supply chain [3, 4]. Wasted food is often linked to wasted resources, such as the land, water, and energy inputs to food production [4–6]. Food waste in school nutrition programs has received increasing attention since the mandate of the USA’s Healthy, Hunger-Free Kids Act (HHFKA) [7], but evidence shows waste rates have not increased under HHFKA nutrition standards [8, 9]. Even still, an average of 21–45% of each school meal is wasted [8–11] among the 30 million children participating in the U.S. National School Lunch Program [12], underscoring the missed opportunity for children to consume healthy food and mitigate waste.

Since food consumption and food waste are corollary behaviors, many schools have turned to the concept of “nudging” to improve students’ eating (and wasting) behaviors in the school lunchroom environment. Thaler and Sunstein (2008) define a nudge as “any aspect of the choice architecture that alters people’s behavior predictably without forbidding any options or significantly changing their economic incentives” [13] , [p. 6]. The concept of nudging is grounded in behavioral economics, which argues that consumers are susceptible to behavioral biases because they often make decisions based on mental shortcuts, or heuristics, which can subsequently lead to sub-optimal (in this case, less healthy or more wasteful) decisions [14–17]. In the case of food choices, for example, research has shown that consumers exhibit present-biased preferences, meaning they place more weight on immediate benefits (e.g., better taste, convenience) relative to delayed benefits (e.g., improved health) when making food decisions [14–16]. Also, consumers can be susceptible to visceral factors, such as sights or smells, when they are in a “hot” state such as being hungry or under stress [15, 16]. Behavioral economists contend that nudging can be used to help consumers overcome these biases and ultimately improve food choice. In the context of the school lunchroom environment, nudges could include tactics like slicing fruit to make it more convenient for students to eat, re-naming fruit and vegetable dishes to make them more appealing, or identifying white milk as the ‘featured’ milk to encourage white milk as the default choice over chocolate milk.

Intervention strategies based in behavioral economics dominate school nutrition research and practice. In the U.S., nudges are a key component of the Smarter Lunchrooms Movement, which combines insights from behavioral economics, psychology, and marketing in an effort to reduce waste while improving student selection and consumption of vegetables and other healthy foods. Over 29,000 U.S. schools have implemented behavioral nudge interventions targeting school meals [18]. Yet, recent critiques have questioned whether their effects on consumer behavior are meaningful or overstated [19, 20]. There are also significant gaps in knowledge related to the long-term impacts of nudges used in the school meal environment. Furthermore, school meal research generally does not take the influence of other important system factors, like the time available to eat [21], menu [22], or day of the week [23], into account when evaluating behavioral nudges. The complexity of school meal environments and the interrelated problems of food waste and dietary behavior require a systems approach that is missing from the available evidence on school meal nudges.

There have been two other literature reviews [24, 25] published on environmental approaches to improving the school nutrition environment, but both of them included interventions that are not nudges, such as changing nutrition policies [25], food pricing [24, 25], and/or providing incentives for healthy behavior [24]. Thus, there is no systematic assessment of the impact of behavioral nudges on school meal dietary behaviors. The aim of this study is to conduct a systematic review to determine the range and quality of available evidence of school meal nudges on student eating behaviors, such as school meal participation, food selection, consumption, and waste.

## Methods

### Search strategy

The systematic review protocol was based on the PRISMA (Preferred Reporting Items for Systematic Review and Meta-Analysis) guidelines [26]. The PICO-C (population, intervention, comparison, outcomes, context) framework was established a priori to determine the inclusion and exclusion criteria for the study. Population consisted of school children in grades Kindergarten - 12 (primary and secondary school students); Intervention had to focus on a classic nudge implemented in the cafeteria environment or marketing and promotion campaigns; eligible comparison groups included intervention versus post-intervention, control versus intervention, and exposed versus unexposed groups; Outcomes included sales, selection, consumption, waste, and school meal participation; Context required studies to be conducted during school lunch or breakfast in the cafeteria. Articles were retrieved from three databases (PubMed, Psych Info, and Web of Science) from June through August 2018 using search terms developed by the authors after a review of 35 known articles and review of the recommendations included on the Smarter Lunchrooms Scorecard [27]. The search terms were divided into two groups. The first included all possible combinations of 1) School lunch, school breakfast, school food, school nutrition, school cafeteria, or school canteen 2) Label*, nudg*, behavioral economics, choice architecture, marketing, environment*, promot*, atmosphere, placement, chef, default option, or slic* and 3) Intake, choice, select*, consum*, waste, sales, or participation. Group two was limited to 1) Smarter Lunchroom* and combined with 2) intake, choice, select*, consum*, waste, sales, or participation. Additionally, articles were excluded if they were 1) not in English and 2) were any format other than a published peer-reviewed paper. There were no exclusion criteria related to publication date. After duplicates were removed, two undergraduate research assistants reviewed the title and abstract to determine if PICO-C criteria were met; a faculty member resolved any disagreement between the research assistants. Articles were downloaded for full-text review if PICO-C criteria were satisfied. The references for each article that underwent full-text review were searched to identify additional papers.

### Data extraction

Targeted data extracted from the studies, using a standardized data extraction form, included: author(s), publication date, study setting, purpose and design, sample characteristics, intervention components and characteristics, data collection methods, intervention outcomes, assessment of intervention integrity, and study implications. For these studies, only data from intervention components that met PICO-C criteria were extracted. Each data extraction was performed initially by an author and verified for accuracy by a second author. A third author was consulted whenever unanimous agreements could not be reached by the first two authors. Studies that included both nudge intervention components and additional intervention components (such as classroom components) that did not meet PICO-C criteria were only included in the review if they used study designs that quantified the specific impact of a nudge in isolation. In these cases, only the data about the nudge intervention and control group were extracted.

### Data synthesis

Meta-analysis could not be performed, as there was extensive heterogeneity among study designs, samples, intervention components, outcome measures and measurement techniques. A narrative synthesis was used to examine the interventions included in the review to describe themes and limitations present in the existing body of literature, and assess the overall quality of published nudge interventions. Study characteristics, intervention components, outcomes and effectiveness were summarized and tabulated to provide an overview of the studies included in the review. For discussion purposes, the nudge internventions for the included studies were reviewed and then classified into three categories of nudge interventions: 1) placement or convenience, 2) marketing or promotion, or 3) variety or portion sizes of foods. Studies that included two or more intervention components were considered “multi-component” interventions. Stata 15 MP software was used to conduct all descriptive statistical analyses.

### Quality assessment

Six criteria including study design, sampling, measurement techniques and methods, intervention integrity checks, confounders, and environmental/systems controls, from a modified version of the Effective Public Health Practice Project’s Quality Assessment Tool for Quantitative Studies [28], were used to assess the quality of each study. Each criterion received a quality score of one, two, or three, and criterion quality scores were summed to yield global quality scores of strong, moderate, or weak for individual studies. Only studies that were of strong or moderate quality were included in final analyses. Quality score criteria and possible point ranges are listed in Table 1.

**Table 1:** Study quality criteria and scores

Criterion of Study Quality	Mean (SD) Observed Range			
	Weak Studies (*n* = 9)	Moderate Studies (*n* = 17)	Strong Studies (*n* = 3)	Full Sample (*n* = 29)
**1). Study Design:** Was there a comparison group and were treatments randomized?	**1.22 (0.42)** 1–2	**2.53 (0.61)** 1–3	**2.67 (0.47)** 2–3	**2.14 (0.82)** 1–3
**2). Sampling Methods:** Were schools and participants selected randomly or through convenience sampling?	**2.00 (0)** 2	**2.00 (0)** 2	**2.00 (0)** 2	**2.00 (0)** 2
**3). Data Collection Methods/Measurement Techniques:** Were valid and reliable methods used to collect outcome data?	**2.56 (0.50)** 2–3	**2.53 (0.50)** 2–3	**3.00 (0)** 3	**2.59 (0.49)** 2–3
**4). Intervention Integrity Checks:** Did the study include any announced or unannounced intervention integrity checks?	**1 (0)** 1	**1.35 (0.48)** 1–2	**2.33 (0.94)** 1–3	**1.34 (0.60)** 1–3
**5). Demographic Confounders Controlled for:** Were relevant demographic confounders (gender, SES, race/ethnicity) controlled for in study design or analysis?	**1 (0)** 1	**2.12 (0.83)** 1–3	**3.00 (0)** 3	**1.86 (0.90)** 1–3
**6). Environmental/Systems Confounders:** Were environmental/systems confounders controlled for in study design or the analysis?	**1.44 (0.50)** 1–2	**1.76 (0.73)** 1–3	**2.00 (0.82)** 1–3	**1.69 (0.70)** 1–3
**Total Study Quality Score** (sum of items 1–6)	**9.22 (0.63)** 8–10	**12.29 (1.02)** 11–14	**15.00 (0)** 15	**11.62 (1.99)** 8–15

*Note*. The total possible study quality score ranged from 6 to 18. Scores for each study quality criterion were assigned based on whether the study exhibited strong (3), moderate (2), or weak (1) characteristics in each category. Studies with scores from 15 to 18 were categorized as strong (*n* = 3), scores from 11 to 14 were categorized as moderate (*n* = 17), and scores from 6 to 10 were categorized as weak (*n* = 9). Environmental/systems confounders included controlling for menu, time available to eat, and/or day of the week, and specifying if salad bar/self-service options were present

## Results

### Study selection

**Fig. 1:**
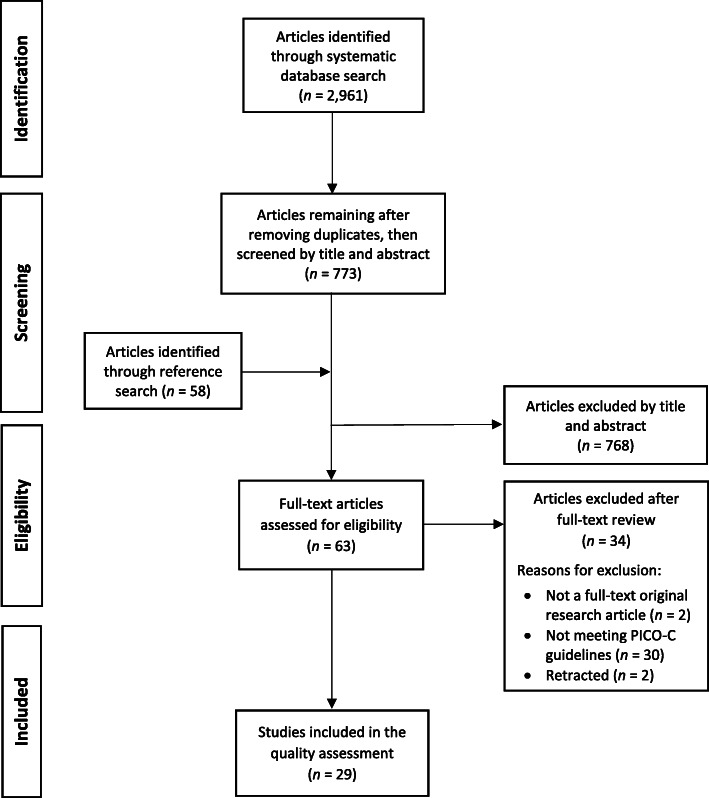
PRISMA flow diagram

Figure 1 depicts the study search and selection process. The systematic database search yielded 2961 studies. After removing duplicate articles, and identifying 58 additional studies through reference search, 768 articles were excluded by title and abstract screening. Among the 63 articles assessed for eligibility, 34 were excluded for not being full-text original research articles, not meeting PICO-C guidelines, or for having been retracted since initial identification. Twenty-nine articles met the selection criteria and were included in the quality assessment.

### Study characteristics

All studies included in the systematic review were conducted in developed countries, including the United States (*n* = 26), France (*n* = 1), Australia (*n* = 1), and the United Kingdom (*n* = 1). Sample sizes for included studies ranged from 25 students to approximately 19,000 students and from one to 21 schools (*n* = 4 did not report sample sizes). Of the 29 studies, 20 (69%) included elementary (primary) school students (grades K-5), 15 (52%) included middle school students (grades 6–8), and 7 (24%) included high school students (grades 9–12), with 13 studies (45%) including participants across multiple age groups. Reported eligibility rates for the Free and Reduced Price Lunch Program (FRL) ranged from less than 10 to 100% (*n* = 7 did not report FRL eligibility rates). Study length ranged from 1 day to three academic years (*n* = 2 did not report study length). Table 2 summarizes included studies.

**Table 2:** Summary of school meal nudge studies (*n* = 29) characteristics and outcomes

Authors, Date [Reference Number] Study Design Study Length Sample Details Quality Rating	Intervention Component(s) (Component Duration)	Outcome Categories: Measurement Technique (Number of Data Collection Days)	Outcome Results
**Adams et al., 2005** [23] Cross-Sectional Study Study Length: 1 day Location: California Age Group: Grades 1–5 *n* = NR, 4 schools Quality Rating: Moderate	− Compared schools with existing salad bars to schools without salad bars (Duration NR)	Selection: Direct Weighing Individual (1 day)	⊘ No significant difference in selection of fruits and vegetables at schools with and without salad bars (NS)
		Consumption: Direct Weighing Individual (1 day)	⊘ No significant difference in consumption of fruits and vegetables at schools with and without salad bars (NS)
**Adams et al., 2016** [29] Cross-Sectional Study Study Length: 1 day Location: Arizona Age Group: Middle School *n* = 533 students, 6 schools Quality Rating: Moderate	− Changing placement of salad bar to inside serving line, instead of outside the serving line (Duration NR)	Selection: Direct Weighing Individual (6 days)	↑ Students self-served 5.38 times (95% CI: 4.04–7.17) more fresh fruits and vegetables by weight when the salad bar was inside the lunch line (141.2 g) compared to outside (26.6 g)
		Consumption: Direct Weighing Individual (6 days)	↑ Students were 4.82 times (95% CI: 3.40–6.81) more likely to consume more fresh fruits and vegetables when the salad bar was inside the lunch line (74.1 g) compared to outside (16.6 g)
		Waste: Direct Weighing Individual (6 days)	↑ Students wasted more fruits and vegetables when the salad bar was inside the lunch line (43%, 29.9 g) compared to outside (12%, 11.7 g) (*p* = NR)
**Cohen et al., 2015** [30] Cluster Randomized Trial Study Length: 10 months Location: Massachusetts Age Group: Grades 1–8 *n* = 1587 students, 10 schools Quality Rating: Moderate	− Using attractive bowls or baskets − Signage and images promoting fruits and vegetables − Changing fruit and vegetable placement (All components 4 months)	Selection: Visual Observation (6 days)	↑ Fruit selection was greater at intervention schools (54.3%) than control schools (51.1%) (OR: 1.45, 95% CI: 1.13–1.87) ↑ Vegetable selection was greater at intervention schools (33.7%) than control schools (50.6%) (OR: 1.91, 95% CI: 1.46–2.50) ⊘ No significant difference in milk selection between intervention and control schools (NS)
		Consumption: Direct Weighing Individual (6 days)	⊘ No significant difference in fruit consumption between intervention and control schools (NS) ⊘ No significant difference in vegetable consumption between intervention and control schools (NS) ⊘ No significant difference in milk consumption between intervention and control schools (NS)
**Conklin et al., 2005** [31] Non-Randomized Controlled Trial Study Length: 12 weeks Location: Pennsylvania Age Group: Grades 9–12 *n* = NR, 6 schools Quality Rating: Moderate	− Nutrition Facts labeling at point of sale for entrées (6 weeks)	Selection: Sales Data (12 weeks)	↑ Decrease in the number of servings selected for pepperoni pizza, cheeseburgers, bacon cheeseburgers, and chicken dishes with > 20 g of fat per serving (*p* < .05) ↑ Increase in the number of servings selected for cheese pizza, hamburgers, and veggie burgers (*p* < .05)
**Delaney et al., 2017** [32] Cluster Randomized Trial Study Length: 4 months Location: Australia Age Group: Grades K – 6 *n* = 2714 students, 10 schools Quality Rating: Moderate	− Increasing the number of food choices/variety − Creative, descriptive names to market food items (non-student developed) − Online pre-orders − Stop Light Labeling − Positive prompting and reinforcement for healthy choices (All components 2 months)	Selection: Sales Data (4 months)	↓ Fewer calories in selected lunches among intervention (1104 kJ) compared to control (1565 kJ) participants at post-intervention (*p* < .001) ↓ Less saturated fat in selected lunches among intervention (2.87 g) compared to control (4.84 g) participants at post-intervention (*p* < .001) ↓ Less sodium in selected lunches among intervention (400 mg) compared to control (527 mg) participants at post-intervention (*p* < .001) ⊘ No significant difference in sugar content of selected lunches between intervention and control at post-intervention (NS)
**Elbel et al., 2015** [33] Non-Randomized Controlled Trial Study Length: 10 months Location: New York Age Group: Grades K – 12 *n* = 19,000 students, 19 schools Quality Rating: Moderate	− Introduction of water jet (Approximately 10 months)	Selection: Visual Observation (4 days)	↑ Three months after implementation, there was a larger increase in water selection among intervention (+ 23%) schools compared to control schools (+ 2%) (*p* < .001) ↑ Ten months after implementation, there was a larger increase in water selection among intervention (+ 22%) schools compared to control schools (− 2%) (*p* < .001)
**Elsbernd et al., 2016** [34] Crossover Study Design Study Length: 12 weeks Location: Minnesota Age Group: Grades K – 5 *n* = 800 students, 4 schools Quality Rating: Moderate	− Verbal prompts for healthy item selection and/or consumption − Serving vegetables first in isolation (All components 3 days)	Selection: Sales Data (5 days)	↑ Selection of peppers was greater on the intervention days (65%) when vegetables were served first in isolation compared to control days (8%) (*p* < .0001)
		Consumption: Visual Observation (5 days)	↑ Consumption of peppers was greater on the intervention days (4.1 g) when vegetables were served first in isolation compared to control days (mean 1.4 g) (*p* < .0001)
		Waste: Visual Observation (5 days)	↑ Waste of peppers was greater on the intervention days (53–64%) when vegetables were served first in isolation compared to control days (8–38%) (*p* = NR)
**Ensaff et al., 2015** [35] Non-Randomized Controlled Trial Study Length: 2 academic years Location: United Kingdom Age Group: Middle & High School *n* = 2200 students, 2 schools Quality Rating: Strong	− Changing food placement − Making plant-based foods more convenient (disposable pots/trays used to serve meals, prefilled pots/trays) − Promotional material (smiley stickers on packaging and posters, end of shelf label, encouraging posters) (All components 6 weeks)	Selection: Sales Data (378 days)	↑ Students were 2.5 times more likely (OR: 2.49, 95% CI: 2.03–3.06) to select the designated items (whole fruit, fruit salad, vegetarian specials, and sandwiches containing salad) during the intervention period than at baseline (*p* < .001) ↑ Students were 3 times as likely to choose a fruit, vegetable, or salad item during the intervention relative to baseline (OR: 3.04, 95% CI: 2.50–3.69) (*p* < .001)
**Goto et al., 2013** [36] Cluster Randomized Trial Study Length: 1 month (approx.) Location: California Age Group: Grades 1–6 *n* = 677 students, 3 schools Quality Rating: Moderate	− Treatment 1: Increased white milk quantity available in the milk cooler compared to chocolate milk − Treatment 2: Students must request chocolate milk (Both conditions 1 week)	Selection: Visual Observation (10 days)	↑ At treatment 1 school (increased milk quantity), selection of white milk increased by 18% between pre- (30%, 74 students) to post-intervention (48%, 118 students) (*p* < .001) ⊘ No significant change in selection of white milk in treatment 2 school (ask for chocolate) (NS)
		Consumption: Direct Weighing Individual (30 days)	⊘ No significant change in white milk consumption at either treatment school (NS)
**Greene et al., 2017** [37] Cluster Randomized Trial Study Length: 9 weeks Location: New York Age Group: Grades 5–8 *n* = 2108 students, 10 schools Quality Rating: Moderate	− Fruit placed first on lunch line − Served two fruit options instead of one − Slicing fruit − Using attractive bowls for whole fruits − Creative, descriptive names to market fruit items (non-student developed) − Fruit “factoids” (promotions) (All components 6 weeks)	Selection: Visual Observation (Average of 5 baseline and 4 intervention days per school)	↑ Increase in average fruit selection from pre- (.59 servings) to post-intervention (.80 servings) (*p* < .001) ↑ Increase in average vegetable selection from pre- (.67 servings) to post-intervention (.98 servings) (*p* < .001) ↑ Increase in average white milk selection from pre- (.10 servings) to post-intervention (.14 servings) (*p* < .001)
		Consumption: Visual Observation (Average of 5 baseline and 4 intervention days per school)	↑Increase in average fruit consumption from pre- (.73 servings) to post-intervention (.83 servings) (*p* < .001) ↑ Increase in average vegetable consumption from pre- (.57 servings) to post-intervention (.86 servings) (*p* < .001) ⊘ No change in average white milk consumption from pre- to post-intervention (NS)
**Hakim et al., 2013** [38] Non-Controlled Trial Study Length: 2 weeks (approx.) Location: Kansas Age Group: Grades K – 8 *n* = 2064 students, 1 school Quality Rating: Weak	− Increasing the number of fruit and vegetable choices and variety offered each day (1 month)	Consumption: Direct Weighing Individual (20 days)	↑ Fruit consumption increased between pre-intervention (40%) and post-intervention (67%) (*p* < .01) ↑ Vegetable consumption increased between pre-intervention (23%) and post-intervention (41%) (*p* < .01)
**Hanks et al., 2012** [39] Before-After Study Study Length: 3 months Location: New York Age Group: High School *n* = 362 students, 1 school Quality Rating: Weak	− Created a convenience line in which only options considered healthy (sub sandwich bar, salad bar, vegetables, whole fruit, fruit parfait, flavored milks) were offered (2 months)	Selection: Self-Reported Survey with no validity or reliability information provided (4 days)	↑ Increase in selection of healthy foods (salad bar, vegetables, fruit, fruit parfaits, and sub sandwiches) from pre- (mean .66 items) to post-intervention (mean .79 items) (*p* < .001) ↑ Selection of flavored milk increased from pre- (mean 0.74 items) to post-intervention (mean 0.85 items) (*p* < .001) ⊘ No significant change in plain milk selection (NS)
		Consumption: Direct Weighing Individual (4 days)	↓ Consumption of less healthy foods decreased by 27.9% (*p* < .001)
**Hanks et al., 2013** [40] Before-After Study Study Length: 4 months Location: New York Age Group: Grades 7–12 *n* = NR, 2 schools Quality Rating: Weak	− Using attractive bowls or baskets − Changing food placement − Creative, descriptive names to market food items (non-student developed) − Verbal prompts to promote healthy items − Creating healthy convenience line with only submarine sandwiches, fruit and vegetable sides (All components 2 months)	Selection: Sales Data (12 days)	↑ Students selecting fruits increased from pre- (47.3%) to post-intervention (53.7%) (*p* = .012) ↑ Students selecting vegetables increased from pre- (35.8%) to post-intervention (44.0%) (*p* < .001)
**Hanks et al., 2016** [41] Cluster Randomized Trial Study Length: 6 weeks Location: New York Age Group: Elementary School *n* = 1133 students, 10 schools Quality Rating: Moderate	− Treatment 1: Branded vegetable characters featured on vinyl promotional banners − Treatment 2: Television promotional segments − Treatment 3: Branded vegetable characters featured on vinyl promotional banners and television promotional segments (All conditions 4 weeks)	Selection: Sales Data (6 weeks)	↑ Increase in average daily vegetable and salad servings selected from pre- (60 servings) to post-intervention (185 servings) in treatment 3 schools (*p* = .028) ⊘ No significant changes from pre- to post-intervention in vegetable and salad servings selected in treatment 1 and treatment 2 schools (NS)
**Hunsberger et al., 2014** [42] Non-Controlled Trial Study Length: 2 months Location: Oregon Age Group: Grades 6–8 *n* = 531 students, 1 school Quality Rating: Weak	− Calorie labels at point of purchase (1 month)	Consumption: Direct Weighing Aggregate (Number of days NR)	↓ Decrease in calories consumed between pre- (668 Kcal) and post-intervention (621 Kcal) (*p* = .0040) ↓ Decrease in fat consumed between pre- (23.1 g) and post-intervention (21.1 g) (*p* = .0025)
**Johnson et al., 2017** [43] Cross-Sectional Study Study Length: NR Location: Louisiana Age Group: Grades 7–12 *n* = 718 students, 21 schools Quality Rating: Weak	− Compared schools with existing salad bars to schools without salad bars (Duration NR)	Consumption: Self-Administered 24-Hour Dietary Recall with no validity or reliability information provided (Number of days NR)	↑ Students in salad bar schools had significantly higher median energy consumption during lunch (452 kcal) than students in non-salad bar schools (395 kcal) (*p* = .0136) ↓ Less fruit was consumed in salad bar schools (.06 cups) than non-salad bar schools (0.25 cups) (*p* = .0061) ⊘ No significant differences in vegetable consumption (NS)
**Kenney et al., 2015** [44] Cluster Randomized Trial Study Length: 2 months Location: Massachusetts Age Group: Elementary, Middle, and High School *n* = NR, 10 schools Quality Rating: Moderate	− Promotional signage highlighting water source locations and promoting consumption (16–23 days) − Cup dispensers located next to cafeteria water fountains (5–22 days)	Selection: Visual Observation (95 days)	↑ Larger increase from pre- to post-intervention in students who take free water during lunch period among intervention (+ 7.3%) compared to control (− 2.0%) (*p* < .001)
		Consumption: Visual Observation (95 days)	↑ Larger increase from pre- to post-intervention in water consumption among intervention (+ 0.53 oz) compared to control (− 0.06 oz) (*p* < .001)
**Larson et al., 2018** [45] Cluster Randomized Trial Study Length: 3 academic years Location: Minnesota Age Group: Grades 9–12 *n* = 364 students, 8 schools Quality Rating: Strong	− Implementing a grab-and-go breakfast cart before school (1 year intervention)	Participation: Administrative Data (2 years baseline and 1 year intervention data)	↑ Participation in school breakfast program increased from 13.0% in the first baseline year (T1) to 22.6% in the intervention year (T3) (*p* = 0.03)
**Miller et al., 2015** [46] Crossover Study Design Study Length: 4 months (approx.) Location: Minnesota Age Group: Grades K – 5 *n* = 758 students, 1 school Quality Rating: Moderate	− Provided increased portions sizes for orange wedges, baby carrots, greens beans, and applesauce as the default portion size (Duration NR)	Selection: Direct Weighing Individual (3 days)	↑ Selection of oranges was higher on intervention days (51%) compared to control days (35%) (*p* < .0001) ↓ Selection of applesauce was lower on intervention days (36%) compared to control days (46%) (*p* < .0001) ⊘ No significant difference in green bean selection (NS)
		Consumption: Direct Weighing Individual (3 days)	↑ Consumption of applesauce was greater on intervention days (119 g) compared to control (77 g) (*p* < .001) ↑ Consumption of orange slices was greater on intervention days (37 g) compared to control (22 g) (*p* < .001) ↑ Consumption of carrots was greater on intervention days (33 g) compared to control (20 g) (*p* = .02) ⊘ No significant difference in green bean consumption
		Waste: Direct Weighing Individual (3 days)	↑ Waste of fruits and vegetables was 11–125% greater on intervention days compared to control days (*p* = NR)
**Miller et al., 2016** [47] Non-Randomized Controlled Trial Study Length: 4 weeks Location: Florida Age Group: Grades 4–7 *n* = 169 students, 1 combined elementary and middle school Quality Rating: Moderate	− Treatment 1: Pre-orders − Treatment 2: Pre-orders with behavioral nudge (message alert if the student did not select a complete meal) (All conditions 2 weeks)	Selection: Video Recorded Observation (6 weeks)	↑ Increases in selection of vegetables from pre- to post-intervention were significant for treatment 1 (pre-orders only, + 17.6%, *p* < .001) and treatment 2 (pre-order with behavioral nudge, + 24.6%, *p* < .001), but not control (+ 5.4%, *p* = NS) ↑ Increases in selection of fruits from pre- to post-intervention were significant for treatment 1 (pre-orders only, + 23.0%, *p* < .001) and treatment 2 schools (pre-order with behavioral nudge, + 37.8%, *p* < .001) but not control (+ 4.8%, *p* = NS) ↑ Increases in selection of low-fat milk from pre- to post-intervention were significant for treatment 1 (+ 7.3%, *p* = .011), treatment 2 (+ 12.2%, *p* < .001), and control (+ 20.7%, *p* = .011)
**Moreno-Black et al., 2017** [48] Repeated Cross-Sectional (Longitudinal) Study Length: 10 months (approx.) Location: Oregon Age Group: Grades 1–5 *n* = 3000 students, 7 schools Quality Rating: Moderate	− Fruit and vegetable placement on salad bar vs hot line (Spring and Fall semester)	Selection: Digital Photography (Number of days NR)	↑ Students were more likely to select fruit if when it was placed on the hot lunch line (*p* < .05) ↑ Students were more likely to select any vegetable when fruit was placed on the hot lunch line (*p* < .001) ↑ Students were more likely to select at least one healthy vegetable item when both fruit (*p* < .001) and vegetables (*p* < .05) were placed on the hot lunch line
**Morizet et al., 2012** [49] Non-Randomized Controlled Trial Study Length: 2 days Location: France Age Group: Elementary and Middle School *n* = 227 students, 3 schools Quality Rating: Weak	− Treatment 1: Basic descriptive label (i.e., new carrot recipe) − Treatment 2: Model-related label (i.e., new carrot recipe, Special Mix for Super Heroes) − Control Group: No labels (All conditions 2 days)	Selection: Visual Observation (6 days)	↑ Selection of new vegetable dish was greater for treatment 1 (*p* = .012) and treatment 2 (*p* = .002) compared to control ⊘ No significant difference between treatment 1 and treatment 2 in selection of familiar and new dishes (NS)
**Redden et al., 2015** [50] Crossover Study Design Study Length: 3 months Location: USA Age Group: Grades K – 5 *n* = 755 students, 1 school Quality Rating: Strong	− Offering youth vegetables in a small cup prior to moving through the lunch line, instead of only serving vegetables through the lunch line (Field Study: 1 control day, 1 intervention day, 3 months apart) (Longitudinal: 1 control/baseline day, 3 intervention days, 1 control day, over 3 month period)	Selection: Visual Observation (Field Study: 2 days, Longitudinal: 5 days)	⊘ Field Study: Likelihood of selecting carrots from lunch line did not differ between treatment and control days (NS) ↓ Longitudinal: Percentage of students selecting broccoli from lunch line was higher on the baseline control day (13.8%) than the first treatment day (4.1%, *p* < .0001), the second (9.4%, *p* < .01), and the third treatment day (5.3%, *p* < .0001) ↓ Longitudinal: Percentage of students selecting broccoli from lunch line higher on final control day (8.5%) than first (4.1%, *p* < .01) and third (5.3%, *p* < .01), but not the second treatment day (9.4%, NS)
		Consumption: Direct Weighing Individual (Field Study: 2 days, Longitudinal: 5 days)	↑ Field Study: Students consumed 10.3 g more carrots total on treatment day (12.7 g) than control day (2.4 g) (*p* < .0001) ↑ Longitudinal: Students consumed more broccoli on the first treatment day (3.99 g), second treatment day (4.06 g) and the third treatment day (2.10 g) than both baseline control (0.84 g) and final control (0.90 g) days (*p* < .0001 for all treatment-control comparisons)
**Schwartz et al., 2007** [51] Cluster Randomized Trial Study Length: 2 months (approx.) Location: Connecticut Age Group: Grades 1–4 *n* = 323 students, 2 schools Quality Rating: Moderate	− Verbal prompts promoting healthy items (Duration NR)	Selection: Visual Observation (2 days)	↑ Students were more likely (OR: 1.9, 95% CI: 1.1–3.3) to select a serving of fruit in the intervention school (48%) compared to the control school (32%)
		Consumption: Visual Observation (2 days)	↑ Students who selected fruit were more likely (OR: 2.3, 95% CI: 1.3–4.2) to consume fruit in the intervention school (87%) compared to the control school (65%)
**Siegel et al., 2015** [52] Non-Controlled Trial Study Length: 4 months Location: Ohio Age Group: Grades K – 6 *n* = 297 students, 1 school Quality Rating: Weak	− Smiley face signs placed next to encouraged items (7 weeks) − Intermittent verbal reminders (Intermittently over 7 weeks)	Selection: Sales Data (Number of days NR)	↑ Increased plain white fat free milk selection from 7.4 to 17.9% of students (*p* < .001) ↓ Decrease in low-fat chocolate milk selection from 86.5 to 77.1% of students (*p* < .001) ⊘ No significant change in overall milk purchases (NS) ⊘ No significant change in fruit purchases (NS)
**Swanson et al., 2009** [53] Non-Controlled Trial Study Length: NR Location: Kentucky Age Group: Grades K – 4 *n* = 491 students, 1 school Quality Rating: Weak	− Slicing apples and oranges before serving (1 day intervention condition, 1 day control condition)	Selection: Digital Photography (2 days)	↑ Selection of sliced oranges (16.2, 95% CI: 13.0–19.7) was greater than selection of whole oranges (5.5, 95% CI: 3.6–7.9) ⊘ No significant difference in apple selection (NS)
		Consumption: Digital Photography (2 days)	↑ More students consumed entire serving of sliced oranges (10.2, 95% CI: 7.6–13.3) than whole oranges (2.3, 95% CI: 1.1–4.0) ⊘ No significant difference in apple consumption (NS)
**Thompson et al., 2017** [54] Non-Controlled Trial Study Length: 8 months Location: Minnesota Age Group: Grades 1–4 *n* = 1861 students, 2 schools Quality Rating: Weak	− Slicing apples − Using attractive bowls or baskets − Changing fruit and vegetable placement − Creative, descriptive names to market food items (non-student developed) − Menu displays with serving size suggestions (All components 4 months)	Selection: Direct Weighing Individual (4 days)	↑ Percent of students selecting a serving of fruit increased from 95.5 to 98.1% (*p* = .02) ⊘ No significant change in vegetable selection (NS)
		Consumption: Direct Weighing Individual (4 days)	⊘ No significant change in fruit consumption (NS) ⊘ No significant change in vegetable consumption (NS)
**Wansink et al., 2013** [55] Cluster Randomized Trial Study Length: 2 weeks (approx.) Location: New York Age Group: Middle School *n =* 643 students, 6 schools Quality Rating: Moderate	− Slicing apples (Duration NR)	Selection: Visual Observation (4 days)	↑ Intervention schools with fruit slicers had a larger increase in average daily apple sales (10%) compared to control schools (6%) (*p* < .01)
		Consumption: Visual Observation (4 days)	⊘ No significant changes in total apple consumption from pre- to post-intervention at treatment schools (NS)
		Waste: Visual Observation (4 days)	⊘ No significant difference in total apple waste (by weight) from pre- to post-intervention at treatment schools (NS)
**Zellner et al., 2016** [56] Non-Controlled Trial Study Length: 2 months Location: Pennsylvania Age Group: Grades 3–4 *n* = 25 students, 1 school Quality Rating: Moderate	− Offering fruit at the end of the meal, instead of during the meal (1 day intervention condition, 1 day control condition)	Consumption: Visual Observation (2 days)	↑ Larger percent of students ate at least some of the vegetable (kale salad) in intervention (100%) compared to control (40%) (*p* = .0017)

*Note. NR* not reported. *NS p*-value not significant. This table only includes descriptions and outcomes for school meal nudge intervention components. Studies which included components that were not nudge interventions, but used methods that allowed nudge components and outcomes to be isolated are included

### Study quality

The results of the quality assessment are displayed in Table 1. The global quality ratings of three studies (10%) were of strong quality [35,45,50), seventeen studies (59%) were of moderate quality [23, 29–34, 36, 37, 41, 44, 46–48, 51, 55, 56], and nine studies (31%) were of weak quality [38–40, 42, 43, 49, 52–54].

Twelve studies (41%) used strong study designs, including nine cluster randomized trials [30, 32, 36, 37, 41, 44, 45, 51, 55] and three crossover study designs [34, 46, 50]. Nine studies (31%) used moderately strong study designs, including five non-randomized controlled trials [31, 33, 35, 47, 49] and four cross-sectional studies [23, 29, 43, 48]. Eight studies (28%) used weak study designs, including six non-controlled trials [38, 42, 52–54, 56] and two before-after studies [39, 40]. All included studies (*n =* 29) used moderate quality sampling methods. None of the studies randomly selected schools, but three randomly selected individual participants within schools [23, 29, 38], and the remaining twenty-five sampled all participants who were eligible for the study within the schools.

Data collection methods were generally strong (*n* = 17), and many studies (*n* = 15) measured multiple outcomes. Strong measurement methods consisted of valid and reliable data collection techniques for each outcome. The most common strong data collection techniques for each outcome category were as follows: participation – administrative data (*n =* 1), selection – sales data (*n =* 7), consumption – individual direct weighing (*n* = 9), and waste – individual direct weighing (*n =* 2). Seventeen studies (59%) used strong data collection methods [23, 29–32, 35, 38, 41, 42, 45–48, 50, 52–54] Zero studies used weak data collection methods. Intervention integrity checks were uncommon among included studies. Only two studies (7%) used strong intervention integrity check methods [35, 45], which consisted of unannounced researcher observations. Six studies (21%) used moderately strong intervention integrity check methods [23, 30, 32, 37, 44, 48], such as announced/planned researcher observations or self-reported intervention integrity checks. The remaining 21 studies (74%) did not report using any intervention integrity or fidelity monitoring and were rated as weak in this category.

Ten studies (37%) used strong methods to address potential confounders (gender, socioeconomic status, and race/ethnicity) and either had no important differences between groups at baseline or controlled for all important differences between groups or participants [29, 30, 32, 33, 35, 36, 45, 47, 50, 51]. Five studies (17%) used moderately strong methods to address potential confounders and controlled for some (but not all) potential confounders at the individual level, or controlled for confounders at the school level [37, 44, 48, 55, 56]. Fourteen studies (48%) used weak methods to address potential confounders and controlled for none of the potential confounders or did not provide information on demographics or differences between groups [23, 31, 34, 38–43, 46, 49, 52–54]. Few studies controlled for or reported on systems and environmental-level factors (specifying if salad bar/self-service options were present, and controlling for menu, time available to eat, and/or day of the week). Zero studies included pre-consumer waste assessments; this systems level factor was not included in the calculation of quality scores for this category. Four used strong methods to address systems factors [23, 29, 50, 56], twelve studies used moderately strong methods [31, 33–35, 37–39, 41, 43, 46, 48, 54], and thirteen studies used weak methods to address systems factors [30, 32, 36, 40, 42, 44, 45, 47, 49, 51–53, 55].

### Summary of intervention components and study outcomes

**Fig. 2:**
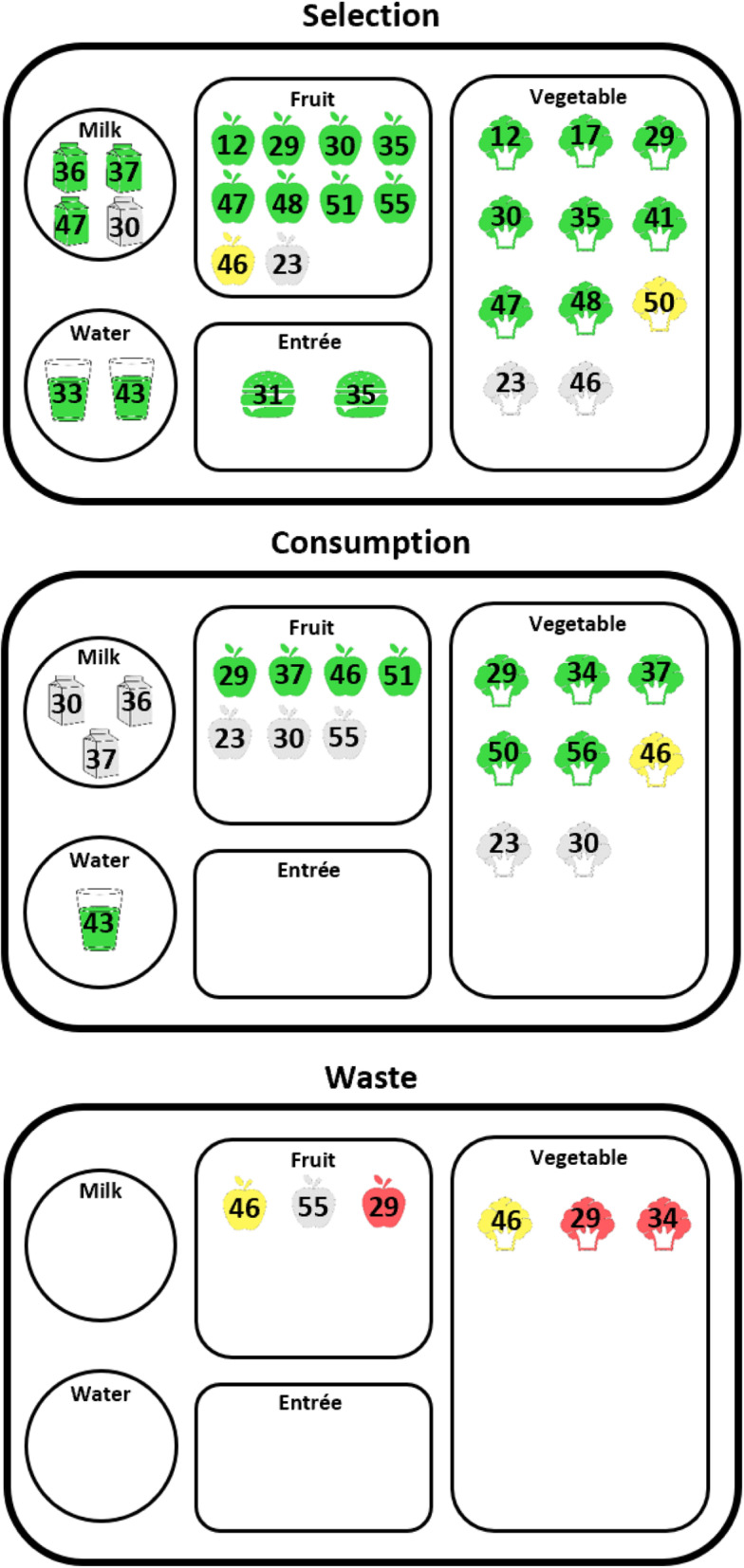
Outcomes from included school meal nudge studies (*n* = 20) listed by school meal component. *Note.* Positive (green), mixed (yellow), null (grey), and negative (red) outcomes are displayed for each meal component category (vegetables, fruits, entrée, milk, and water). One study [45] (not displayed in this figure) also measured participation in school meals. Only three studies were ranked as high quality [35, 45, 50]. All others were of moderate quality. Outcomes for studies in the following categories 1) placement or convenience, 2) marketing or promotion, 3) variety or portions, and 4) multi-component studies appear in Supplemental Figure 1

The rest of the results section will exclusively discuss results from studies of strong and moderate quality. Eighteen studies measured selection, twelve studies measured consumption, and four studies measured waste of school lunch foods. As shown in Fig. 2, fruits and vegetables were the predominant meal components examined across studies. One study [45] measured student participation in school breakfast. Studies are grouped for discussion based on intervention type (placement or convenience; marketing or promotion; variety or portion sizes of foods). Studies that included two or more intervention components were considered to be multi-component studies and are discussed separately. Studies with multiple components that fall in the same category [36, 41] will be included in both multi-component and specific intervention type sections.

#### Multi-component studies

The most common intervention component used in multi-component interventions (*n =* 9, 45% of included studies) was changing the placement of food or milk [30, 35–37]. Multi-component intervention study designs included six cluster randomized trials [30, 32, 36, 37, 41, 44], one crossover study [34], and two non-randomized controlled trials [35, 47]. Outcomes of multi-component studies were largely positive (73%), but there were also more null outcomes (23%) than in most other intervention categories (see Supplemental Figure 1). However, only two of the multi-component studies (23%) used study designs or statistical methods that allowed authors to separate effects for each intervention component [36, 41]. The large proportion (77%) of multi-component studies whose effects cannot be analyzed separately by intervention component limits our ability to draw strong conclusions about the impact of individual intervention components. The rest of this section will only provide detailed results for strong studies and multi-component studies with designs that allow for isolating effects.

Two (22%) of the nine multi-component studies used different experimental conditions to isolate the effects of each intervention component [36, 41]. Both studies were of moderate quality. Goto and colleagues (2013) [36] implemented an intervention that modified the availability of white and chocolate milk over the course of 1 month (*n =* 3 schools). The three conditions in this intervention included treatment 1: increased white milk quantity available in the milk cooler compared to chocolate milk, and treatment 2: chocolate milk is not on display and students must request chocolate milk to receive it (making it the less convenient option), and control group: no changes were made to milk during school lunch. Selection of white milk increased for participants in treatment 1, but not treatment 2 or the control group (treatment 1: *p* < .001, treatment 2: *p* = .125, control: *p =* 1.00, see Table 2) relative to baseline. There was no significant change in milk consumption relative to baseline in treatment 1, treatment 2, or the control group (treatment 1: *p =* .50, treatment 1: *p =* .89, control: *p =* .69).

Hanks and colleagues (2016) [41] implemented a six-week multi-component study that used branded vegetable characters to promote vegetable and salad selection (*n =* 10 schools). The four study conditions included treatment 1: branded vegetables characters featured on vinyl banners in the cafeteria, treatment 2: television promotional segments with branded vegetable characters shown in the cafeteria, and treatment 3: both vinyl banners and television segments, and a control group that did not receive an intervention. Participants in treatment 3 significantly increased their selection of vegetable and salad servings from pre- to post-intervention (*p* = .028, see Table 2), while participants in treatments 1 and 2 and the control group did not significantly change their vegetable and salad selection (all *ps* = not significant, specific *p*-values not reported).

Of the nine multi-component studies [30, 32, 34–37, 41, 44, 47], one (11%) [35] received a strong quality rating. Ensaff and colleagues’ (2015) [35] multi-component intervention (*n =* 2 schools) was implemented over two academic years and included changing food placement, making plant-based food more convenient (through the use of disposable pots/trays used to serve meals, and prefilled pots/trays), and promotional materials (smiley stickers on packaging and posters, end of shelf labels, encouraging posters) to promote healthy food. Study participants were significantly more likely to select items designated as healthy (fruit, vegetables, vegetarian specials, and sandwiches containing salad) during the intervention compared to baseline (*p* < .001, see Table 2). Despite this study’s strengths, it is not possible to draw conclusions about the effectiveness of individual intervention components.

#### Placement/convenience interventions

Interventions that exclusively fell in the placement/convenience category (*n =* 8, 40% of included studies) included changing food, milk, or salad bar placement [29, 36, 48], changing the timing/order of when foods were served [50, 56], slicing fruit before serving [55], implementing a grab-and-go breakfast cart [45], and introducing water jets [33]. Study designs included three cluster randomized trials [36, 45, 55], one crossover study [50], one non-randomized controlled trial [33], one repeated cross sectional (longitudinal) study [48], one cross sectional study [29], and one non-controlled trial [56].

Of the eight placement/convenience studies [29, 33, 36, 45, 48, 50, 55, 56], two (25%) received strong quality ratings [45, 50]. Larson and colleagues (2018) [45] implemented and evaluated a grab-and-go breakfast cart before school over three academic years (*n =* 8 schools). Participants in the intervention group significantly increased their school breakfast participation relative to baseline (*p* = .003, see Table 2). An intervention by Redden and colleagues (2015) [50] offered students vegetables in a small cup prior to moving through the lunch line (*n =* 1 school). Data was collected over four treatment days and three control days (in which students were not offered extra servings of vegetables) spaced out over a 3 month period. The percent of students who selected carrots did not differ between treatment and control days, and broccoli selection was generally greater on control days (*p* < .001, see Table 2. Nonetheless, participants increased their consumption of both carrots (*p* < .0001) and broccoli (*p* < .0001) on treatment days compared to control days.

Including the studies described above, six placement/convenience studies had selection outcomes [29, 33, 36, 48, 50, 55], five studies had consumption outcomes [29, 36, 50, 55, 56], and two had food waste outcomes [29, 55] (see Supplemental Figure 1). Most studies assessing selection had positive findings (*n* = 7) for vegetables [29, 48], fruits (29,48,55], milk [36], and water [33], while the remaining selection study had a mixed outcome for vegetables [50] Most consumption outcomes were positive (*n* = 4) for vegetables [29, 50, 56] and fruits [29], while two null consumption outcomes were reported for fruits [55] and milk [36]. Most waste outcomes were negative (i.e. increased waste) for fruits (*n* = 1) [29] and vegetables (*n* = 1) [29], while one null waste outcome was reported for fruits [55]. One (previously described) study found increased school breakfast participation [45]. In examining all outcome categories (participation, selection, consumption, and waste) together, interventions with components related to placement/convenience had twelve positive outcomes, one mixed outcome, three null outcomes, and two negative outcomes.

#### Marketing/promotion interventions

Interventions that exclusively fell in the marketing/promotion category (*n =* 3, 15% of studies) included one study that used verbal prompts to encourage healthy item selection and/or consumption [51], one study that used nutrition facts labels at point of sale [31], and one previously described study that used branded vegetable characters on promotional materials [41]. These studies used cluster randomized trials [41, 51] or non-randomized controlled trial designs [31]. All three studies received moderate quality ratings.

Studies in this category had three positive selection outcomes for vegetables [41], fruits [51], and entrées [31]. One positive outcome was reported for fruit consumption [51]. No studies measured food waste nor participation. In examining selection, and consumption together, studies with intervention components related to marketing/promotion had four positive outcomes, and no mixed, null, or negative outcomes (see Supplemental Figure 1).

#### Variety/portions interventions

Interventions that exclusively fell in the variety/portions category (*n* = 2, 10% of studies) included one study that increased portion sizes of fruits and vegetables [46] and one study that implemented salad bars [23]. These studies used crossover [46] and cross-sectional study designs [23]. Selection outcomes included one mixed outcome for fruits [46], and three null outcomes for vegetables [23, 46] and fruits [23]. Consumption outcomes included one positive fruit outcome [46], and two null outcomes for vegetables [23] and fruits [23]. Waste outcomes included two mixed outcomes for vegetables [46] and fruits [46]. In examining all three outcome categories together, studies with intervention components related to variety/portions had one positive outcome, four mixed outcomes, five null outcomes, and no negative outcomes (see Supplemental Figure 1).

## Discussion

In this study, we systematically assessed the relationship between behavioral nudges and student selection, consumption, waste, and meal participation in school cafeteria settings. There were only three behavioral nudge studies rated as strong quality, with seventeen studies rated as moderate quality. Results from the moderate and strong quality studies suggest that behavioral nudges generally have a positive relationship with selection, mixed relationship with consumption, negative relationship with waste (i.e. increased waste), and positive relationship with meal participation. However, there were few studies examining the influence of nudge interventions on meal participation or waste. Yet, waste may be the most important outcome variable since it takes selection and consumption into account, as well as meal participation, indirectly.

Many (*n* = 9, 45% of strong and moderate studies) of the studies included in this review examined nudge interventions with multiple components, most of which evaluated the influence of nudge interventions on selection outcomes. Multi-component and marketing/promotion interventions generally had positive outcomes for selection, and variety/portions had no studies with overall positive selection outcomes. There are not enough studies evaluating the impact of nudge interventions on consumption behaviors to determine which intervention category is most effective for this outcome, but the current evidence does not suggest that there is an advantage to multi-component studies for consumption. None of the intervention categories exhibit a desirable impact on waste outcomes, but there are few studies published on this outcome.

Given that children generally do not meet the recommended intake for fruits and vegetables, health advocates may be most interested in interventions that increase fruit and vegetable consumption. All studies that found significant vegetable consumption increases (*n* = 5) included at least one placement/convenience intervention, and three of these studies incorporated a strategy to provide a portion of the lunch period to focus on vegetable consumption. Both Redden et al. [50] and Elsbernd et al. [34] served vegetables first in isolation, whereas Zellner et al. [56] offered fruit at the end of the meal instead of during the meal. Taken together, these findings suggest that manipulating the order of when vegetables or their competition (which are often fruits) are served has the most evidence of influencing vegetable consumption relative to other nudge strategies. Health advocates may also be particularly interested in interventions that influence milk consumption, since chocolate milk is the only government subsidized sugar-sweetened beverage included in school meals in the US. Yet, there were no single-component studies of strong or moderate quality that found a positive association between nudge interventions and milk consumption. However, there was one study that found a positive increase in water consumption during school lunch [44]. Kenney and colleagues [44] implemented promotional signs to highlight cafeteria water source locations and water consumption, as well as installed cup dispensers next to cafeteria water fountains. Water consumption increased among the intervention group, but decreased among the control group from pre to post. The authors did not examine if this increase in water consumption occurred at the expense of milk consumption, but other research has demonstrated that mealtime increases in water consumption most often replace chocolate milk consumption and may lead to decreases in body mass index [57].

The results of the three studies that exclusively studied marketing/promotions were encouraging as they consistently yielded positive outcomes. While more studies are needed to confirm the efficacy of these types of nudge interventions overall, there were no studies examining the impact of social norming interventions during school meals. Several recent reviews have highlighted how social norms influence eating behaviors across the lifespan [58–60]. For example, Burger et al. found that participants were significantly more likely to select a healthy snack when they had been informed of others’ prior healthy choices [61]. This type of norming intervention would be relatively low cost and easy to implement in the school setting, but more work is needed to ensure these results translate to the school food environment.

**Fig. 3:**
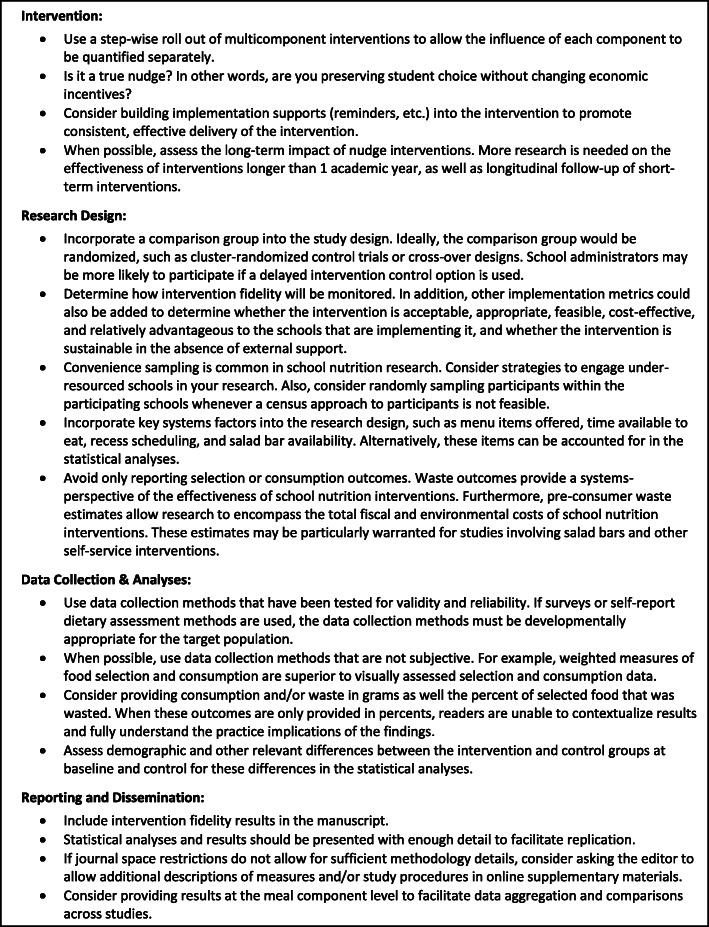
Methodological considerations and recommendations for designing future behavioral nudge interventions in school cafeterias

There were distinct patterns in the quality criteria scores. Even studies of weak overall quality had moderate to strong data collection methods and measurements, suggesting that valid and reliable measurement techniques are likely required for publication. Most strong and moderate studies did not report any assessment of intervention fidelity. None of the included studies randomly sampled schools or school districts. Schools’ focus on academic priorities may increase the likelihood of declining to participate in research studies, making random selection difficult. Also, communities located in close proximity to colleges and universities are likely over sampled due to the need for feasible data collection. Behavioral nudge studies should be considered in the context of the bias created by convenience sampling techniques. In particular, this bias may influence the implementation metrics of behavioral nudges since it is likely that interventions that are acceptable and feasible in a high-resource school may not be so in under-resourced communities. Future studies are needed to consider the efficacy and implementation metrics of behavioral nudges in schools serving high-risk students, as detailed in Fig. 3.

Another key finding of this study was that few papers included relevant environmental and systems factors in the study design. Since there are many factors that influence eating behaviors [62, 63], it is necessary to include external factors in the study design, such as maintaining consistency in the available menu items and time available to eat [21] across study groups and observation dates, to best isolate the relationship between the intervention and outcomes of interest. These details also facilitate study replication and systematic review by other researchers. Omissions regarding the availability of salad bars or other student self-service options make the validity of visual observation methods, such as the quarter waste method, of measuring consumption unclear since these methods are not valid when there is variation in the portions of foods selected [64]. Furthermore, interventions should evaluate efficacy in the context of the overall school meal system since interventions that improve selection or consumption but increase pre or post-consumer waste may not be sustainable given schools’ limited resources and the increased focus on food waste reduction. For example, salad bars are associated with increased pre-consumer waste [65], but none of the studies evaluating salad bar implementation as a method to reduce food waste incorporate pre-consumer waste measurement into the study design. In the interest of intervention sustainability, future salad bar research should incorporate strategies to reduce and measure overall waste instead of only focusing on post-consumer values.

It is also important to note that this review focused on nudge interventions as defined by Thaler and Sunstein; such interventions change the choice environment but do not remove choice or change incentives. Other reviews on the impact of environmental changes on school meal behaviors have included interventions such as removing or banning certain items (e.g., chocolate milk) or changing prices (e.g., reducing prices for healthy options), which do not meet the classical definition of a nudge. Some have argued these tactics, particularly restricting choice, are more effective at changing behavior and are appropriate when we are restricting the choices of children who do not understand the long-term consequences of their choices. Others have pushed back that there is inherent value in preserving choice, though, as consumers, even children, find value in having options. Most of the studies (*n* = 18) included in Driessen et al’s review of environmental changes to improve weight or food behavior outcomes in school lunch settings were policy interventions, such as removing ‘junk’ food from school cafeterias and placing caloric restrictions on the food sold during meals [25]. The authors concluded that district, state, or national policies to modify the school food environment have a positive association with improving child food behaviors, such as reductions in BMI, reductions in unhealthy food purchases, and consuming a lower percentage of energy from fat [25]. However, impacts on vegetable consumption were inconsistent [25], suggesting that policy alone may not be enough to improve children’s consumption of vegetables. These findings, along with those from our current study, suggest that future research consider placement and/or convenience nudges as a complement to new or existing school nutrition policies.

### Limitations

This systematic review has important limitations to consider. Most notably, the variety of outcomes, reporting, and methodologies did not allow us to conduct a meta-analysis, limiting our ability to quantify the overall impact of nudge interventions. In addition, our conclusions were exclusively limited to peer-reviewed literature which could be subject to publication bias. If so, our findings may overestimate the efficacy of school meal nudge interventions since studies finding null or undesirable results are less likely to be published; however, several null and negative findings were included in our systematic review suggesting that this may be a lesser issue. In addition, school meals may not be the most appropriate setting for nudge interventions since, in many countries, strict school nutrition standards ensure that all foods served are relatively healthy compared to other retail settings where there are much less healthy foods available. However, vegetables are differentially wasted in higher amounts in school meals [66] suggesting that some school meal components may still be appropriate nudge targets.

## Conclusions

Based on the evidence identified in this review, school meal nudges are positively associated with selection behaviors and their influence on consumption is unclear. The limited evidence available suggests that nudges are positively linked to meal participation but have an undesirable, positive association with food waste. Placement/convenience nudge interventions have the most evidence to positively influence vegetable consumption, particularly those that provide vegetable isolation opportunities. Our findings also underscore the need for future research on this topic. As noted in Fig. 3, future work should incorporate implementation metrics, isolate the impact of each intervention, and take systems factors into account.

## Additional files

**Additional file 1: Supplemental Figure 1.** Category-specific outcomes from included school meal nudge studies (*n* = 20) listed by school meal component.

**Additional file 2: Supplementary File.** Example article search strategy (*PubMed*).

## Data Availability

The datasets used and/or analyzed during the current study are available from the corresponding author on reasonable request.
