# Supplementary material for: A systematic review of school meal nudge interventions to improve youth food behaviors
Source: Int J Behav Nutr Phys Act. 2020 Jun 19;17:77. doi: 10.1186/s12966-020-00983-y (PMC7304192; doi:10.1186/s12966-020-00983-y)
Supplement: Supplementary file 1 — Additional file 1: Supplemental Figure 1. Category-specific outcomes from included school meal nudge studies (n = 20) listed by school meal component. [file 12966_2020_983_MOESM1_ESM.pdf]

Supplemental Figure 1. Category-specific outcomes from included school meal nudge studies (*n*=20) listed by school meal component

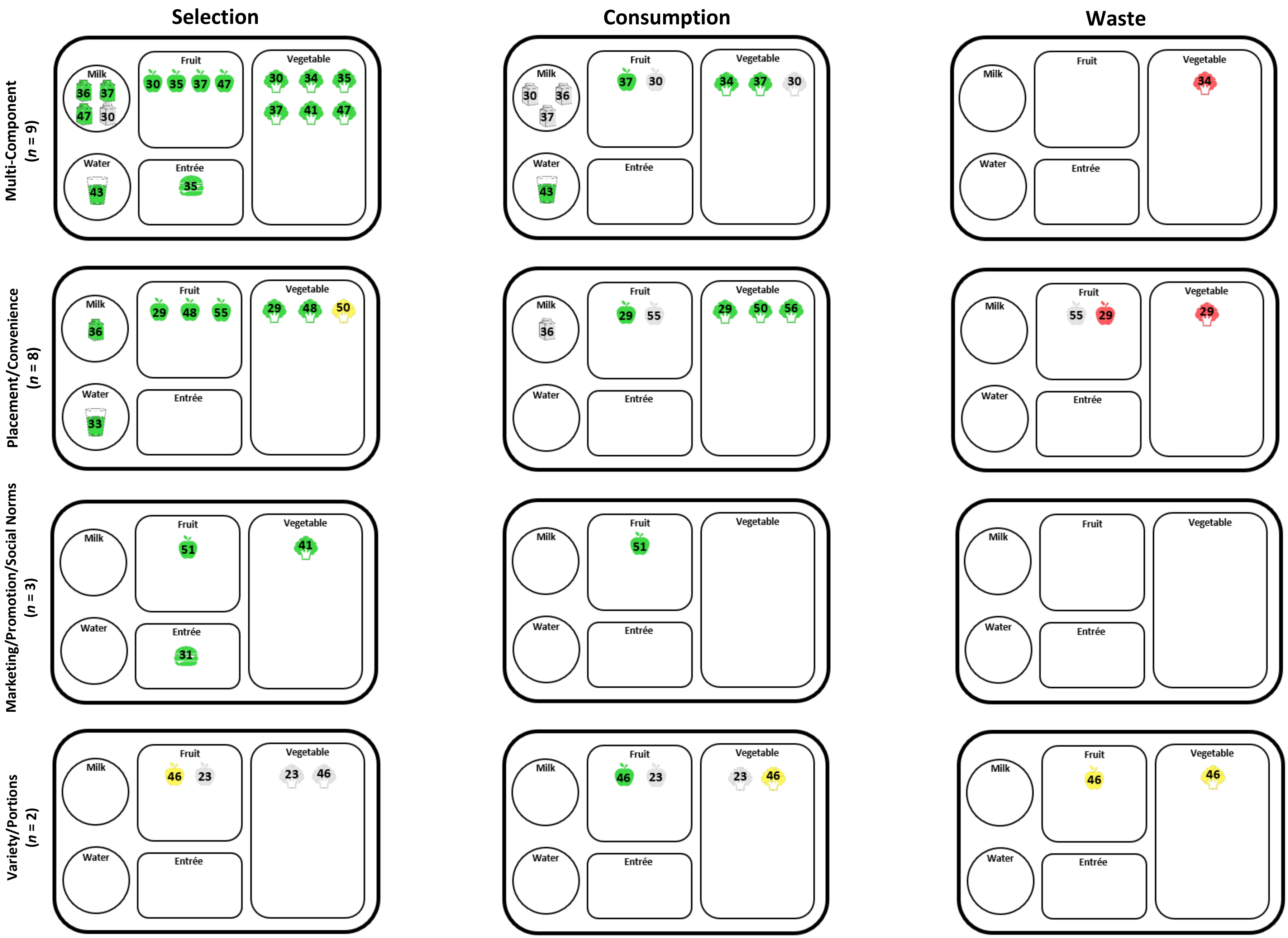

Note. Positive (green), mixed (yellow), null (grey), and negative (red) outcomes are displayed for each meal component category (vegetables, fruits, entrée, milk, and water). There were studies [36, 41] which had multiple components within a single category, and appear on both the multi-component and category specific figures. One study [45] (not displayed in this figure) also measured participation in school meals. Only three studies were ranked as high quality [35, 45, and 50]. All others were of moderate quality.
