# Supplementary material for: A systematic review of school meal nudge interventions to improve youth food behaviors
Source: Int J Behav Nutr Phys Act. 2020 Jun 19;17:77. doi: 10.1186/s12966-020-00983-y (PMC7304192; doi:10.1186/s12966-020-00983-y)
Supplement: Supplementary file 2 — Additional file 2: Supplementary File. Example article search strategy (PubMed). [file 12966_2020_983_MOESM2_ESM.docx]

**Supplementary File: Example search strategy (*PubMed*)**

| **Search Group** | **Search Term** | **Filters or Restrictions** | **Number of Results** |
| --- | --- | --- | --- |
| 1 | (“school lunch” or “school breakfast” or “school food” or “school nutrition” or “school cafeteria” or “school canteen”)  AND  (“label*” or “nudg*” or “behavioral economics” or “choice architecture” or “marketing” or “environment*” or “promot*” or “atmosphere” or “placement” or “chef” or “default option” or “slic*”)  AND  (“intake” or “choice” or “select*” or “consum*” or “waste” or “sales” or “participation”) | English language  Full-text original research article | 286 |
| 2 | (“smarter lunchroom*”)  AND  (“intake” or “choice” or “select*” or “consum*” or “waste” or “sales” or “participation”) | English language  Full-text original research article | 6 |
